# Supplementary material for: Detection of a Novel Gull-like Clade of Newcastle Disease Virus and H3N8 Avian Influenza Virus in the Arctic Region of Russia (Taimyr Peninsula)
Source: Viruses. 2025 Jul 7;17(7):955. doi: 10.3390/v17070955 (PMC12300997; doi:10.3390/v17070955)
Supplement: Supplementary file 1 [file viruses-17-00955-s001.zip › viruses-3688977-supplementary.pdf]

Table S1. Amino acid substitutions in key positions of Influenza virus proteins according to the FluServer (https://flusurver.bii.a-star.edu.sg/).

Protein	Mutations	Reference strain
HA	I4V;L15F;G16S;P20S;L41I;I78R;N97D;K108N;N153S;G160A;S175N;T176A;S209N;W234G;L242Q;S244G;P255S;Q343H;I351X;N501T;V506I;M552I	HA A/HongKong/1/1968(H3N2)
M1		M1 A/Duck/Guangdong/E1/2012(H10N8)
M2		M2 A/Turkey/Ontario/6118/1968(H8N4)
NA	K5Q;I26V;V31I;E40G;K41N;S44V;V49I;K58R;V59I;I63T;I71V;H73Y;I74L;G79E;K125I;I191V;N257K;I262V;I265T;S268N;I301V;V327E;S355T;I376V;G389R;R390K;V392I;M412L;S413T;R415K;Y450H;K451E;V462I	NA A/Quail/Italy/1117/1965(H10N8)
NP	R400K	NP A/Mallard/Astrakhan/263/1982(H14N5)
NS1	D152E	NS1 A/Duck/Alberta/60/1976(H12N5)
NS2	S60N;E67D	NS2 A/Duck/Memphis/546/1974(H11N9)
PA	D272E;E382D;Y535H;I543L;K615R	PA A/Netherlands/219/2003(H7N7)
PB1	K177E;V200I;K360E;S654N;N694S	PB1 A/Duck/HongKong/24/1976(H4N2)
PB2	T105I;V292I;V338I;M444V	PB2 A/Mallard/Astrakhan/263/1982(H14N5)

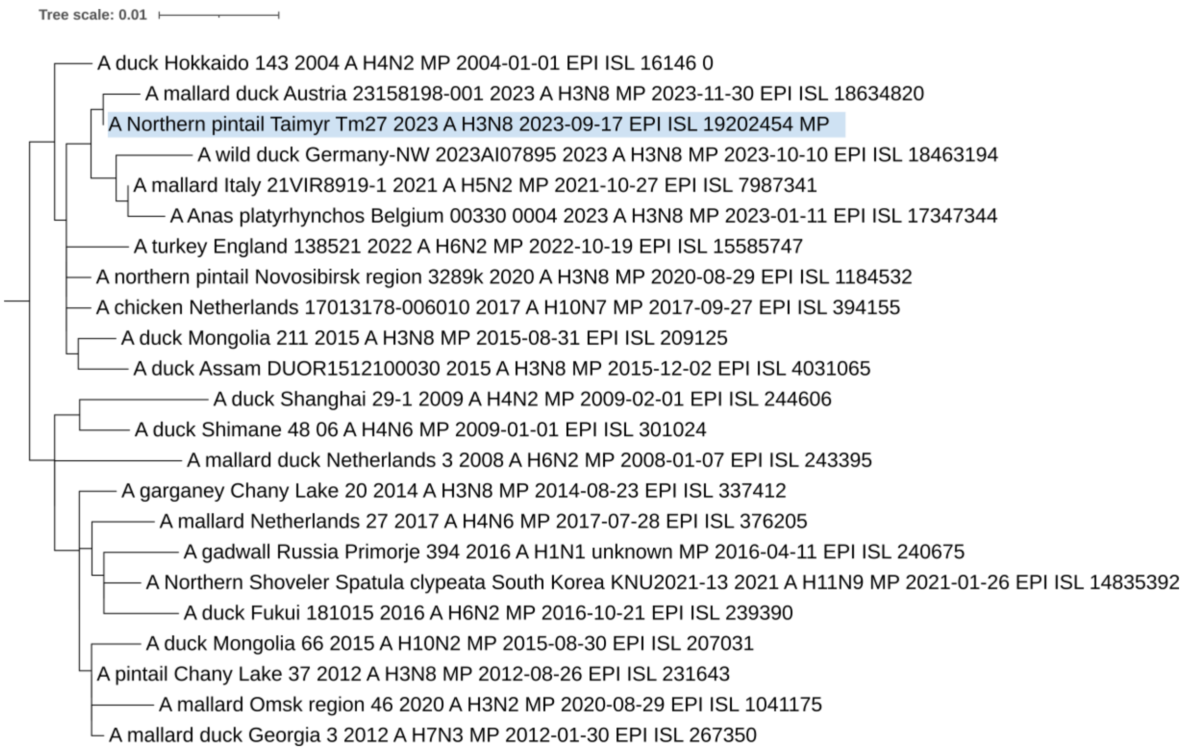

Figure S1. Maximum likelihood phylogenetic tree of the MP genome segment of avian influenza viruses isolated in the Taimyr Peninsula.

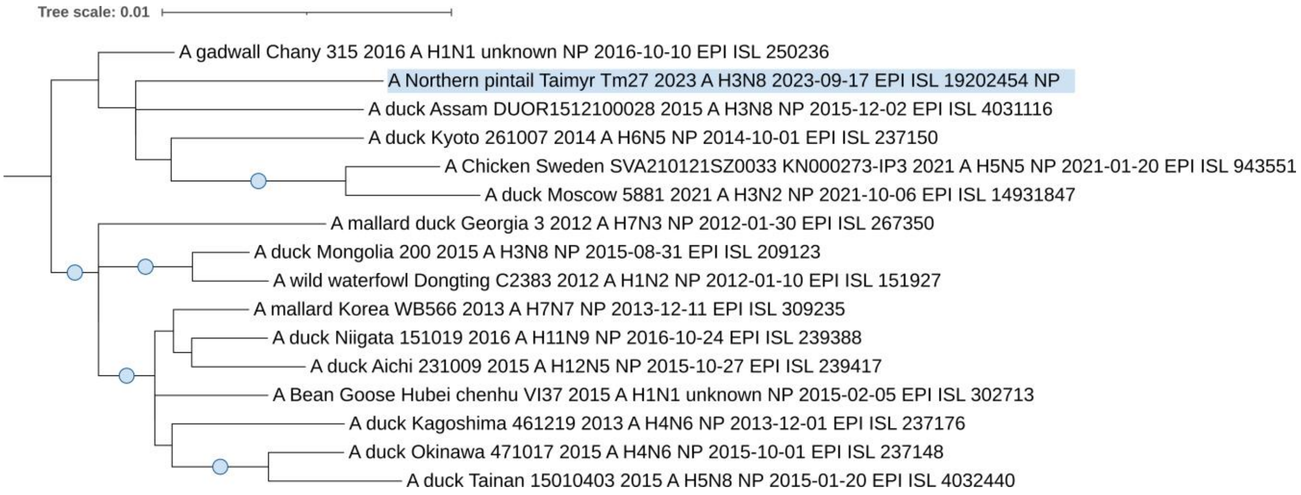

Figure S2. Maximum likelihood phylogenetic tree of the NP genome segment of avian influenza viruses isolated in the Taimyr Peninsula. The blue circle symbol denotes branches with values SH-aLRT > 80% and UFboot > 95%.

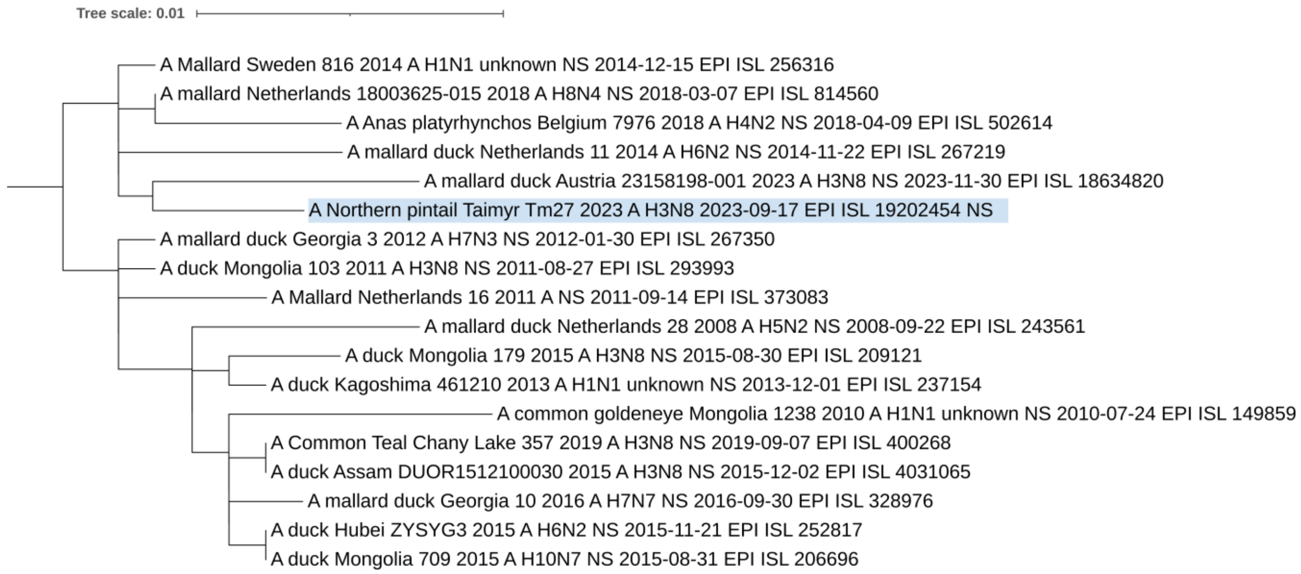

Figure S3. Maximum likelihood phylogenetic tree of the NS genome segment of avian influenza viruses isolated in the Taimyr Peninsula.

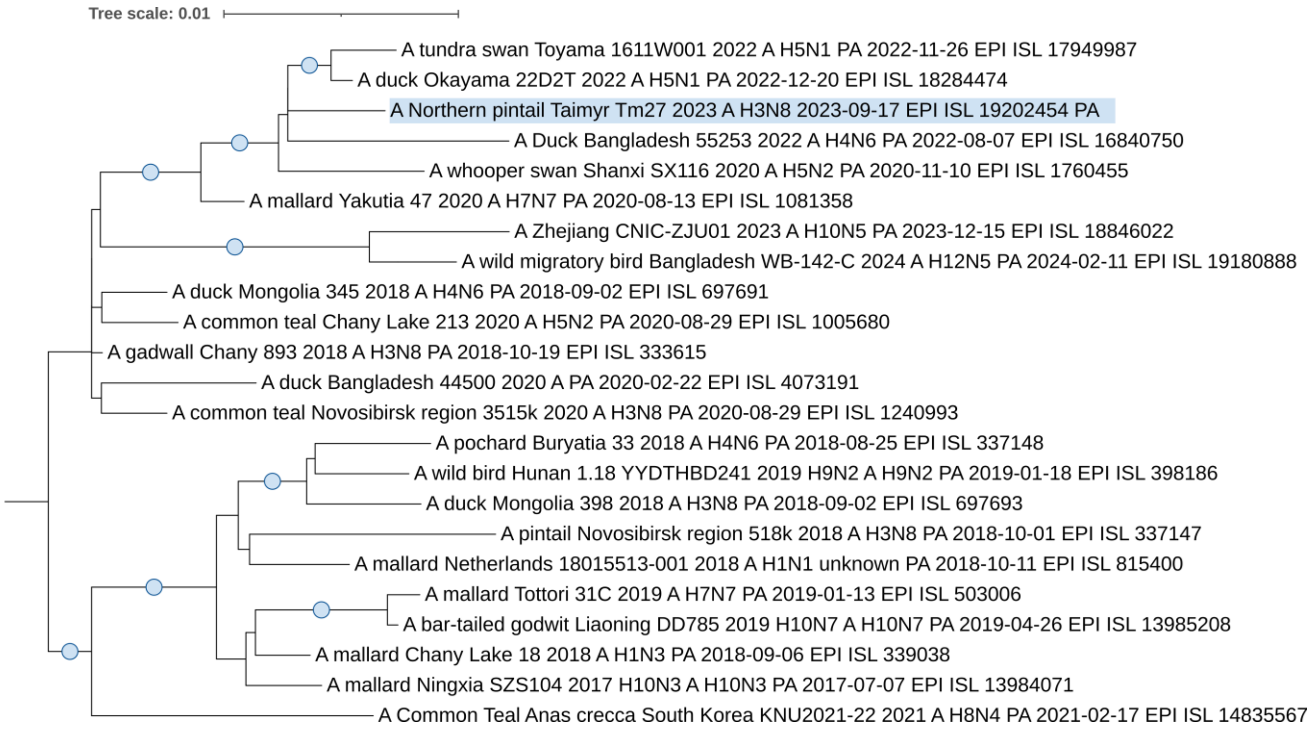

Figure S4. Maximum likelihood phylogenetic tree of the PA genome segment of avian influenza viruses isolated in the Taimyr Peninsula. The blue circle symbol denotes branches with values SH-aLRT > 80% and UFboot > 95%.

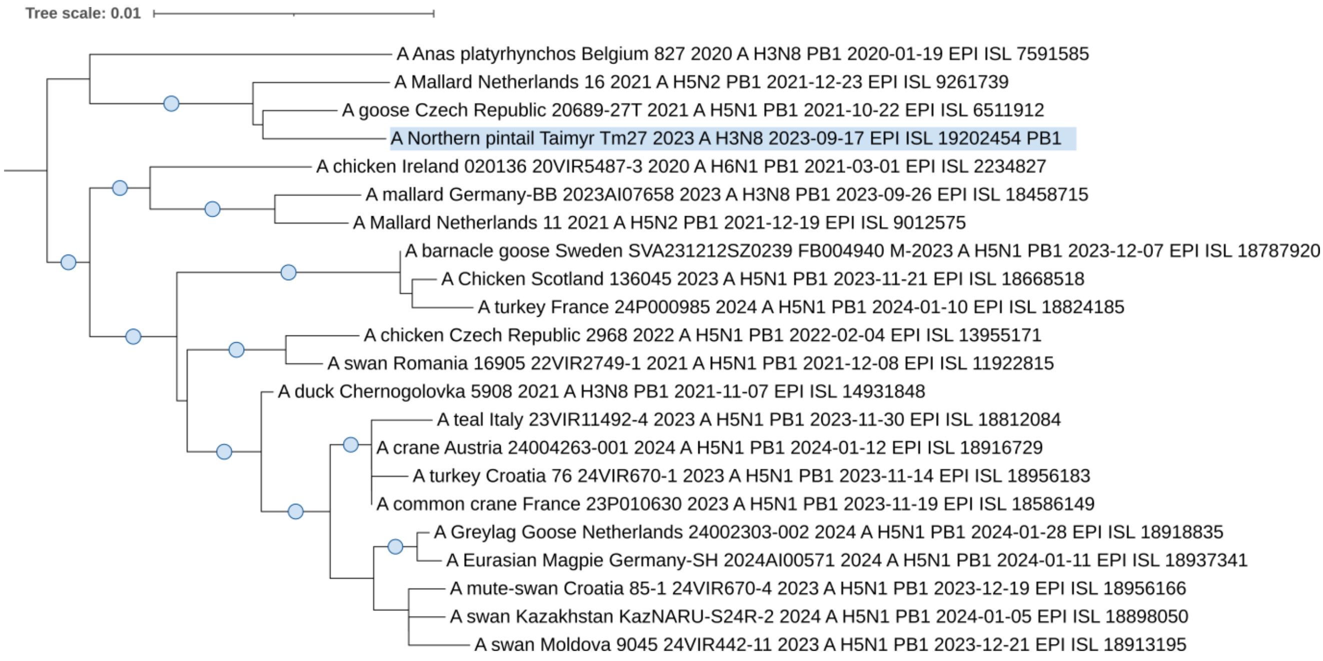

Figure S5. Maximum likelihood phylogenetic tree of the PB1 genome segment of avian influenza viruses isolated in the Taimyr Peninsula. The blue circle symbol denotes branches with values SH-aLRT > 80% and UFboot > 95%.

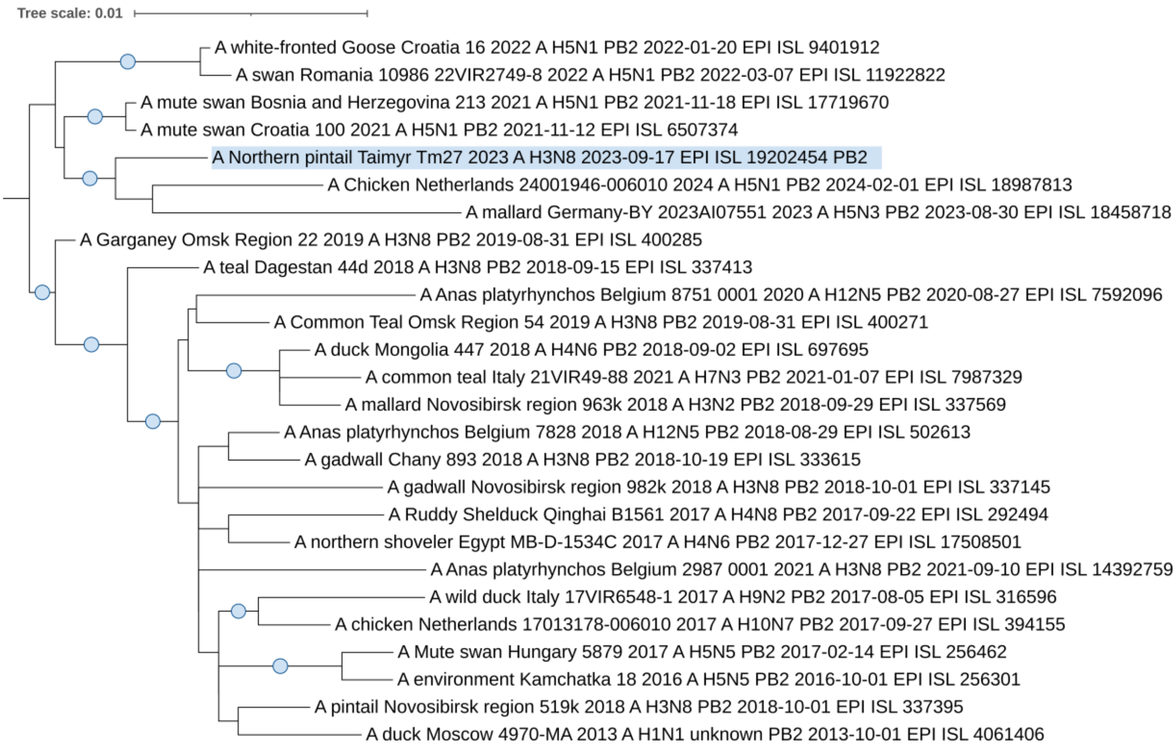

Figure S6. Maximum likelihood phylogenetic tree of the PB2 genome segment of avian influenza viruses isolated in the Taimyr Peninsula. The blue circle symbol denotes branches with values SH-aLRT > 80% and UFboot > 95%.
